# Toward a Census of Bacteria in Soil

**DOI:** 10.1371/journal.pcbi.0020092

**Published:** 2006-07-21

**Authors:** Patrick D Schloss, Jo Handelsman

**Affiliations:** Department of Plant Pathology, University of Wisconsin–Madison, Madison, Wisconsin, United States of America; Stanford University, United States of America

## Abstract

For more than a century, microbiologists have sought to determine the species richness of bacteria in soil, but the extreme complexity and unknown structure of soil microbial communities have obscured the answer. We developed a statistical model that makes the problem of estimating richness statistically accessible by evaluating the characteristics of samples drawn from simulated communities with parametric community distributions. We identified simulated communities with rank-abundance distributions that followed a truncated lognormal distribution whose samples resembled the structure of 16S rRNA gene sequence collections made using Alaskan and Minnesotan soils. The simulated communities constructed based on the distribution of 16S rRNA gene sequences sampled from the Alaskan and Minnesotan soils had a richness of 5,000 and 2,000 operational taxonomic units (OTUs), respectively, where an OTU represents a collection of sequences not more than 3% distant from each other. To sample each of these OTUs in the Alaskan 16S rRNA gene library at least twice, 480,000 sequences would be required; however, to estimate the richness of the simulated communities using nonparametric richness estimators would require only 18,000 sequences. Quantifying the richness of complex environments such as soil is an important step in building an ecological framework. We have shown that generating sufficient sequence data to do so requires less sequencing effort than completely sequencing a bacterial genome.

## Synopsis

Soil is more than dirt. It is the source and sink of nutrients, wastes, pharmaceuticals, and energy required to make Earth supportive of life—it is Earth's most vital organ. Although we know a considerable amount about the physical structure and chemistry of soil, there is a glaring paucity of knowledge regarding the microbial component responsible for its many functions. Over the past 100 years, microbiologists have attempted to characterize the biodiversity of microbial life in soil, and many had reached the unsatisfying conclusion that bacteria may be too diverse to count. Schloss and Handelsman have developed statistical models that they apply to molecular data to predict that the richness of bacteria in 0.5-g soil samples from Alaska and Minnesota were 5,000 and 2,000 species, respectively. At the current level of sampling, approximately 20% of the bacteria appear to be endemic to both soils. The enumeration and description of these organisms points to the need and relative ease of characterizing bacterial communities to identify the organisms responsible for sustaining all of life.

## Introduction

Enumerating the human population of a country or region through a census is an ancient problem that is complicated by the challenges inherent in accurately representing a large and often inaccessible population. The same issues manifest in censuses of microbial communities, but are intensified by greater complexity and methodological challenges. Although a complete census of a country is theoretically possible, it is currently impractical to survey all 10^9^ bacterial cells in a gram of soil [1], making a sample-based census the best option for estimating richness—the number of bacterial taxa in soil. To do so accurately requires a reliable means to access the bacteria, a reasonable definition of “species,” and a robust description of the frequency distribution of the species. Just as a country's census describes a fundamental property of that country, an environment's richness is the most fundamental descriptor of community structure, and patterns of richness can be correlated with an environment's geography, productivity, extremeness, climate change, and degree of isolation [2]. Our inability to estimate richness impedes investigation of the effects of soil chemistry, pollution, and land use on the soil microbial community.

The method used to access the microbial biodiversity assuredly shapes the outcome of a census. Culture-based methods suggest that a gram of soil contains fewer than 100 species [3], but these are undoubtedly underestimates because multiple lines of evidence indicate that fewer than 1% of the species in soil are presently culturable [4]. Culture-independent methods include DNA reassociation and 16S rRNA gene sequencing, which have provided conflicting results due to the problems inherent in defining a species and in estimating the frequency distribution of species in soil. Depending on how the data are analyzed, DNA reassociation experiments produce richness estimates ranging from 4,000 to 10,000,000 genome equivalents per 10 or 30 g of soil [5–11]. The variability in these estimates stems from application of different assumptions to reassociation curves, and their interpretation is complicated by the lack of controls that account for intergenomic variation. Finally, DNA reassociation kinetics cannot be used to compare the membership of different communities.

An alternative method relies on analysis of 16S rRNA gene sequences amplified from soil by PCR [12]. The power of this method lies in its use of the universal tool of bacterial phylogeny and our ability to define operational taxonomic units (OTUs) based on the relatedness of sequences. Estimates of richness have been obtained through parametric or nonparametric empirical models of species frequency distribution to produce richness estimates between 590 and 100,000 species per gram of soil [13–15]. Parametric models have assumed that the incidence of different species follows a lognormal [13], Pareto [16], or uniform distribution [14]. Although the lognormal model has been useful as a “null model” [17], data are insufficient from any soil community to support reliance on a lognormal or Pareto frequency distribution, and we are unaware of any dataset that supports a uniform frequency distribution [18]. Analyses based on nonparametric models, which do not assume a defined frequency distribution but are based on the frequency of abundant community members [19–21], estimate a minimum richness of 590 species based on 16S rRNA gene sequences in a Scottish soil [15,22]. Although the extent of the universality of phylum-specific PCR primers and potential toxicity of some fragments is not well understood, these effects would reduce the perceived richness. Finally, use of 16S rRNA gene sequences permits direct comparisons of the membership of different communities.

Previously, Dunbar et al. [17] modeled the frequency distribution of 16S rRNA genes in four Arizonan soil communities by fitting a lognormal frequency distribution. Using 200 16S rRNA gene fragments from each soil community, this analysis estimated that 10 g of soil contained between 3,000 and 8,000 16S rRNA gene restriction fragment length polymorphism (RFLP) profiles. Previous analysis using the same four libraries found that the similarity of 16S rRNA gene sequences with the same RFLP profile ranged between 52.2% and 99.9% [3], which makes interpretation of the analysis difficult. We were interested in developing this approach further by analyzing large 16S rRNA gene sequence collections that had not been initially screened by RFLP profiling. Our approach was to find a simulated community whose samples resembled our sampling of 1,033 16S rRNA genes from a clone library constructed from a single 0.5-g sample of Alaskan soil. For the purposes of comparison, we also analyzed two large 16S rRNA gene sequence collections that were recently published as part of a soil metagenomic sequencing project, but were not characterized beyond their phylogenetic affiliation [23].

## Results

### Estimating the Bacterial Richness in the Alaskan Soil Library

**Figure 1:**
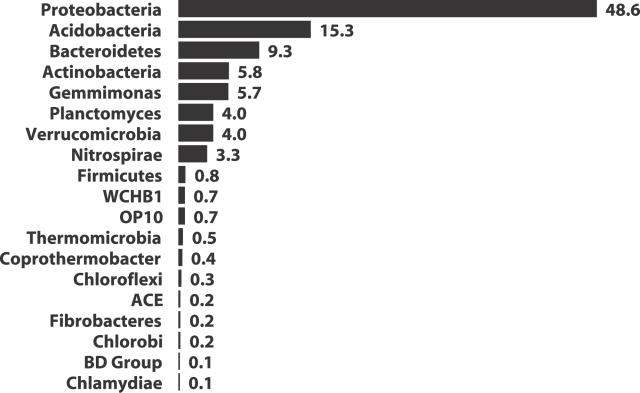
Phylum-Level Delineation of the 16S rRNA Gene Fragments in Alaskan Soil Gene fragments (*n* = 1,033) were isolated and sequenced from an Alaskan soil. Candidate phyla WCHB1, OP10, ACE, and BD Group have no sequenced representatives.

The aim of this work was to estimate the taxonomic richness in an Alaskan soil sample through a library of 16S rRNA gene sequences derived from the sample. We assigned more than 92% of the 1,033 Alaskan 16S rRNA gene sequences to seven phyla, including the Proteobacteria (48.6%), Acidobacteria (15.3%), Bacteroidetes (9.3%), Actinobacteria (5.8%), Gemmimonas (5.7%), Planctomycetes (4.0%), and Verrucomicrobia (4.0%); the remaining sequences clustered within 12 phylum-level delineations, four of which had no cultured members (Figure 1). Each phylum was sampled at least twice, except for candidate phylum BD Group and the phylum Chlamydiae, which were each observed once.

We used furthest neighbor clustering to assign sequences to OTUs based on the pairwise genetic distance between sequences. Although controversial [24], Jukes-Cantor-corrected distances less than 0.03 are considered to correspond to a strain-level delineation, 0.03 to species, 0.05 to genus, 0.15 to class, and 0.30–0.40 to phylum [25–28]. Considering potential intragenomic differences between copies of 16S rRNA genes and errors due to sequencing and alignment [29,30], the 0.03 cutoff is also a pragmatic choice since it probably represents the most stringent OTU definition that is practically obtainable using 16S rRNA genes. Since the intragenomic distance between 16S rRNA gene sequences is typically less than 0.03, at this distance, replicate 16S rRNA gene sequences from the same genome would form a single OTU.

**Figure 2:**
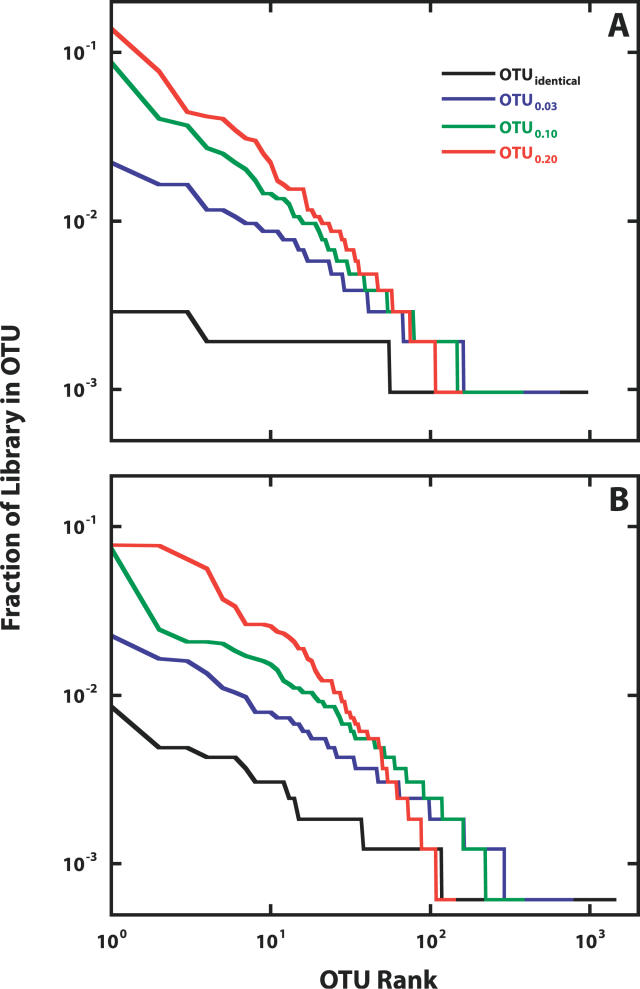
Rank Abundance Plot of the Alaskan and Minnesotan 16S rRNA Gene Libraries Alaskan (*n* = 1,033) (A) and Minnesotan (*n* = 1,633) (B) 16S rRNA gene libraries are plotted and describe the distribution of the 16S rRNA genes among OTUs defined as a group of sequences that are either identical or no more than 3%, 10%, or 20% different.

To simplify the reporting of our results, OTUs will be designated OTU_x.xx_, where the subscript represents the maximum distance between any two sequences within that OTU (Figure 2). In the Alaskan 16S rRNA gene sequence collection containing 1,033 sequences, we observed 633 OTUs_0.03_. We observed 472 OTUs_0.03_ once and 94 OTUs_0.03_ twice (Figure 2A). The three most abundant OTUs_0.03_ affiliated with members of the phylum Gemmimonas (*n* = 23 sequences in the OTU_0.03_ from 19 distinct primary sequences), Duganella sp. (*n* = 17 sequences in the OTU_0.03_ from 13 distinct sequences), and Rhodoferax sp. (*n* = 17 sequences in the OTU_0.03_ from 15 distinct sequences). These three OTUs_0.03_ were not observed in the Minnesotan sequence collection.

Since the most abundant OTU_0.03_ in the Alaskan 16S rRNA gene library was observed only 23 times, we were unable to obtain meaningful fits of parametric frequency distribution models to the OTU_0.03_ frequency distribution [31]. Attempts to identify parameters that would define simulated communities following either a Pareto or uniform frequency distribution resembling the observed distribution were unsuccessful. The predicted abundance of the most abundant OTUs in the Pareto-distributed communities was too high, and the abundance of the rarest OTUs was too low. We successfully simulated the OTU_0.03_ frequency distribution observed in the Alaskan 16S rRNA gene sequence collection by altering the richness and evenness of random frequency distributions using a truncated lognormal frequency distribution (Table 1). The relative abundance of each OTU in the simulated communities that followed the truncated lognormal model was

**Table 1:**
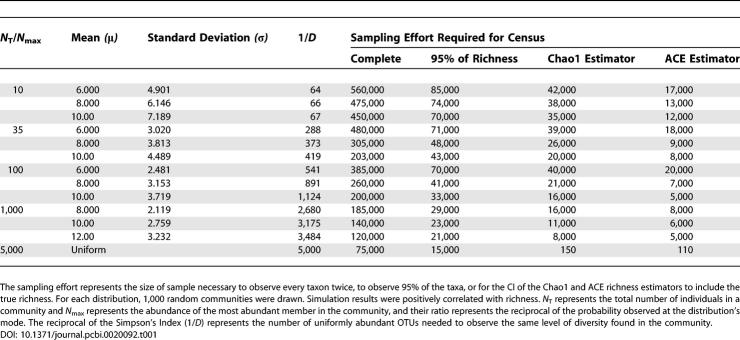
Example of Simulation Results for Lognormal and Uniformly Distributed Communities with a Richness of 5,000


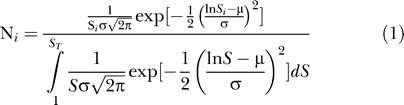


where *S*_i_ is the i^th^ OTU and *N*_i_ is the relative abundance of individuals in that OTU. The maximum possible value of i is the total number of OTUs in the community, *S*_T_. *N*_1_ is the abundance of the most abundant OTU (*N*_max_), and *N*_T_ is the sum of all *N*_i_ values.

**Figure 3:**
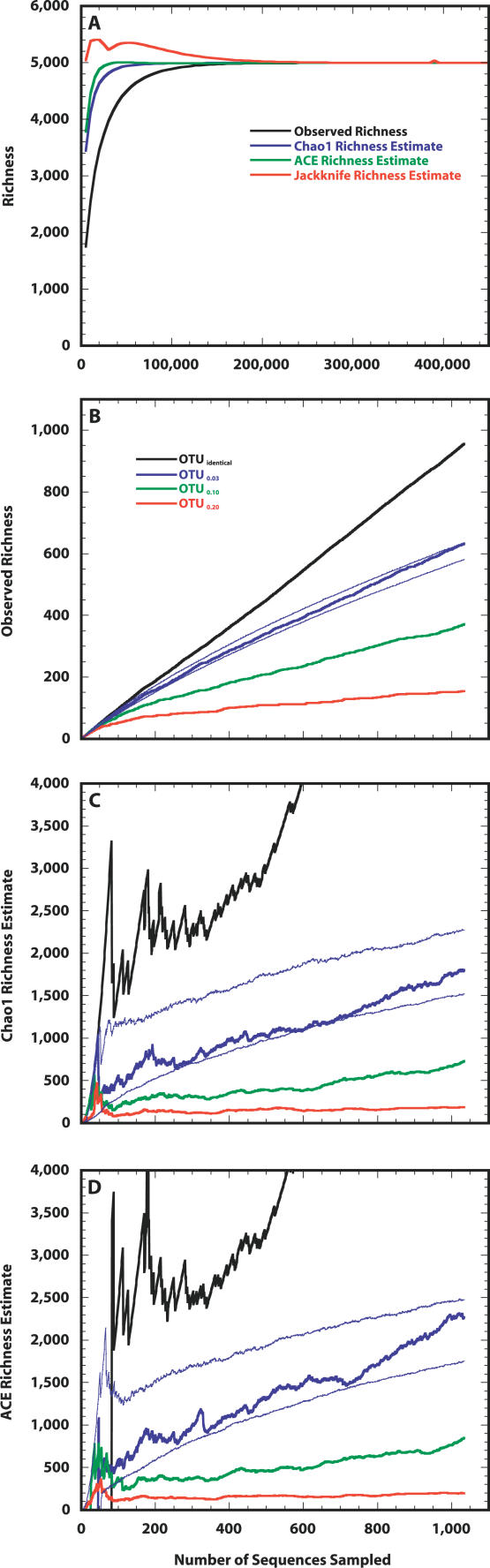
Estimating the Richness of Taxa in the Simulated Alaskan Soil Community (A) Average number of taxa observed and estimated richness for the simulated Alaskan soil community (*S*_T_ = 5,000, μ = 6.000, and σ = 3.020) over the course of randomly sampling 480,000 individuals. (B–D) The distribution of 16S rRNA sequences obtained from Alaskan soil falls within the 95% CI that would have been obtained for the distribution derived from sampling 1,033 individuals from this simulated community as measured using the observed (B) and estimated—Chao1 (C) and ACE (D)—richness. The thin blue lines in (B), (C), and (D) represent the 95% CIs for each metric using the simulated Alaskan soil community.

Next, we heuristically identified the normal mean (*μ* = 6.000), standard deviation (*σ* = 3.020), and OTU_0.03_ richness (*S*_T_ = 5,000) for a truncated lognormal distribution in which the distribution of its samples resembled the distribution observed in the Alaskan sequence collection (Figure 2A and Table 1). Further confirmation for the plausibility of the simulated community was obtained by comparing the percentage of the total community represented by the most abundant OTU (100 × *N*_max_/*N*_T_) in the simulated community (2.9%) to the value observed from the sequence collection (2.2%). These values are comparable to the range 2.9%–8.3% observed by Dunbar et al. [17], but are considerably higher than the range 0.1%–1% suggested by Curtis et al. [13]. We found that the reciprocal of the Simpson's index (1/*D*) for the simulated community was 288, which was similar to the value observed for the sequence collection of 223. The values for 1/*D* are considerably higher than the range 52–107 observed by Dunbar et al. [17]. To sample every OTU in the Alaskan simulated community twice with 95% confidence would require sequencing 480,000 16S rRNA gene fragments, and to observe 95% of the richness, 71,000 16S rRNA genes would be required (Figure 3 and Table 1). To obtain an estimate of the true richness using either the ACE or Chao1 nonparametric richness estimator would require sampling 18,000 or 39,000 16S rRNA genes, respectively, which represented sampling 65% and 85% of the true richness (Figure 3 and Table 1).

Since we were unable to obtain a robust estimate of species richness with our 16S rRNA gene sequence collection without assuming some distribution a priori, we relaxed the OTU definition to obtain a robust nonparametric richness estimate. The OTU_0.20_ richness estimate collector's curves began to stabilize late in sampling (Figure 3). Although additional sampling would improve the precision of the OTU_0.20_ richness estimate, the Chao1 (188.20, 95% confidence interval [CI] 174–212), ACE (200, CI 181–234), and Jackknife (203, CI 184–222) estimates were similar.

### Comparison of Alaskan and Minnesotan Soils Microbial Communities

Recently, the microbial community of a Minnesota farm soil was characterized by metagenomic (direct cloning and analysis of DNA from a soil sample) and 16S rRNA gene sequencing analyses [23]. The authors constructed two separate 16S rRNA gene libraries by using a cell fractionation-based DNA isolation procedure, and sequenced 1,633 overlapping gene fragments from the two libraries [23,32]. We reanalyzed their pooled sequence data to determine the richness of the Minnesota farm soil and to determine the degree of OTU membership that was conserved between the Minnesotan and Alaskan soil communities.

**Figure 4:**
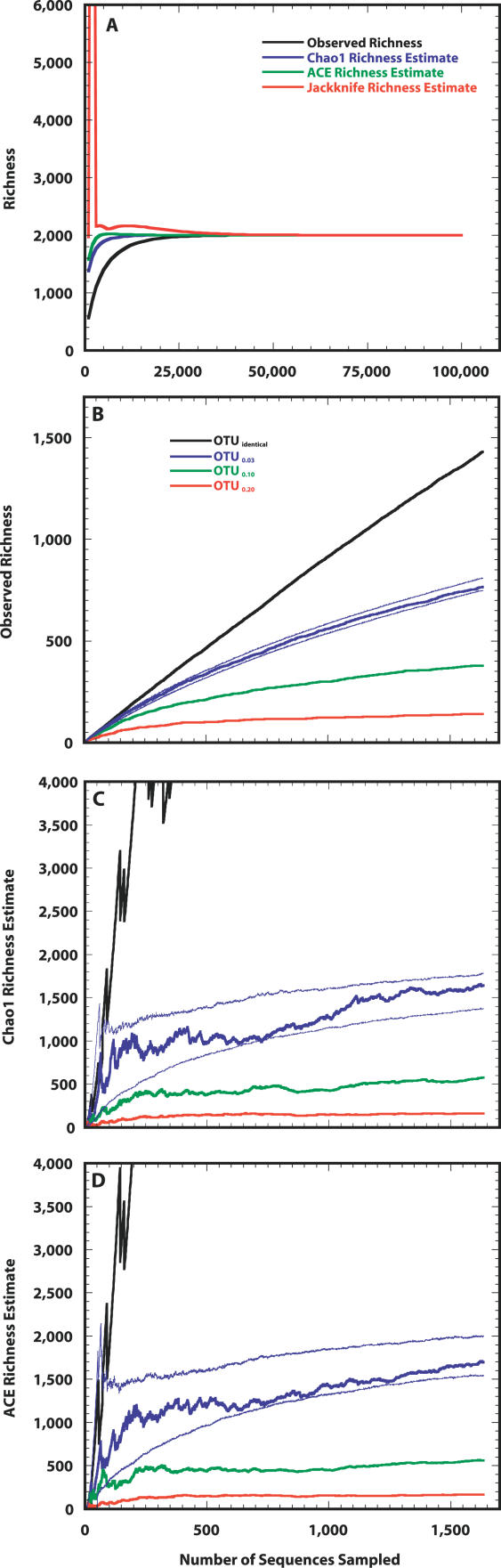
Estimating the Richness of Taxa in the Simulated Minnesotan Soil Community (A) Average number of taxa observed and the estimated richness for the simulated Minnesotan soil community (*S*_T_ = 2,000, *μ* = 8.000, and *σ* = 3.813) over the course of randomly sampling 100,000 individuals. (B–D) The distribution of 16S rRNA gene sequences obtained from the Minnesotan soil falls within the 95% CI that would have been obtained for the distribution derived from sampling 1,633 individuals from this simulated community as measured using the observed (B) and estimated—Chao1 (C) and ACE (D)—richness. The thin blue lines in (B), (C), and (D) represent the 95% CI for each metric using the simulated Minnesotan soil community.

Collector's curves for the number of OTU_0.03_ observed and estimated in the Minnesota soil library were flatter than the Alaskan collector's curves (Figure 4). In the Minnesotan collection, the observed OTU_0.03_ richness was 767, and we observed 477 OTUs_0.03_ once and 128 OTUs_0.03_ twice (Figure 2B). The nonparametric richness estimates were 1,647 (Chao1), 1,704 (ACE), and 2,248 (Jackknife); however, each estimate continued to increase with sampling. The three OTUs_0.03_ most frequently observed in the Minnesotan sequence collection contained 37, 27, and 26 sequences, and each clustered within the phylum Chloroflexi; no representatives of these OTUs_0.03_ were observed in the Alaskan sequence collection.

We identified one simulated community with a truncated lognormal distribution that had a richness of 2,000 (*μ* = 8.000, *σ* = 3.813) whose samples resembled the distribution observed in the Minnesotan sequence collection. The percentage of the clones represented by the most abundant OTU_0.03_ was 2.3%, and it was 3.9% in the simulated community. The simulated community had a higher 1/*D* value than that observed from the sequence data (311 versus 237). The Minnesotan simulated community had a lower richness and more uniform evenness than we observed for the Alaskan simulated community. If the Minnesotan simulated community is a true reflection of the OTU_0.03_ distribution, then we are 95% confident that sequencing of 90,000 16S rRNA genes would result in observing every OTU_0.03_ at least twice. Sequencing 16,000 16S rRNA gene fragments would allow us to observe 95% of the true richness. To obtain a nonparametric estimate of richness through the ACE and Chao1 estimators, 2,000 and 5,500 16S rRNA gene sequences, respectively, would need to be sequenced for the estimate's CI to include 2,000; the CI for the Jackknife estimator already includes 2,000. The sequencing of 2,000 and 5,500 16S rRNA genes would result in observing 43.9% and 72.5% of the true richness, respectively.

**Figure 5:**
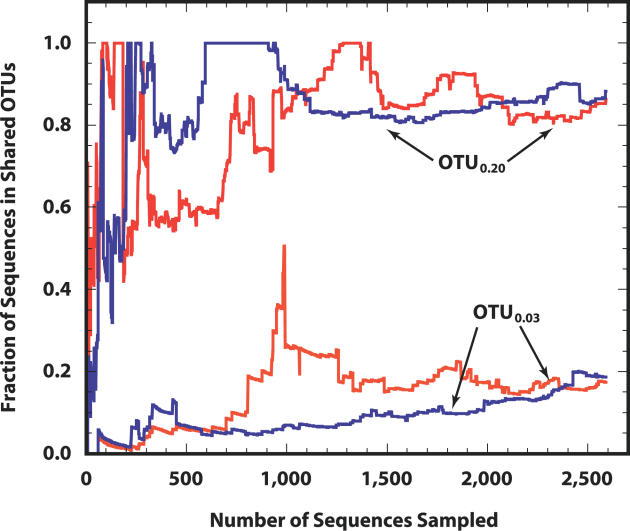
Similarity of Alaskan and Minnesotan Soil Microbial Communities Collector's curves describing the effect of sampling on the estimated fraction of sequences from the Minnesotan (red lines) and Alaskan (blue lines) libraries belonging to shared OTUs_0.03_ and OTUs_0.20_.

It is difficult to determine whether the difference in estimated richness between the Alaskan and Minnesotan simulated communities was due to ecological differences or differences in DNA extraction methods [33] or both. The collector's curve for the estimated fraction of the Minnesotan library shared with the Alaskan library, 0.18 (standard error [SE] = 0.07), indicated that this value is close to the true value and that the fraction of the Alaskan library shared with the Minnesotan library, 0.17 (SE = 0.06), continued to increase with additional sampling (Figure 5A). Our observation that 18% of the sequences in the Minnesotan library belonged to OTUs_0.03_ shared with the Alaskan collection indicates either that a large fraction of these OTUs_0.03_ are endemic to different soils or that the different DNA extraction procedures preferentially lysed a subset of the OTU_0.03_ membership, or both.

Similar to our analysis of the Alaskan 16S rRNA library, when we relaxed the OTU definition to analyze the OTU_0.20_ richness of the Minnesotan 16S rRNA membership, we observed richness estimates that were not sensitive to further sampling. The terminal Chao1 (165, CI 151–197), ACE (169, CI 156–196), and Jackknife (174, CI 158–190) estimates were similar; this is approximately 85% of the OTU_0.20_ richness observed in the Alaskan 16S rRNA library. The fraction of sequences from the Minnesotan library that belonged to OTUs_0.20_ shared between the two libraries was 0.86 (SE = 0.10) and the fraction of sequences from the Alaskan sequences that belonged to OTUs_0.20_ shared between the two libraries was 0.88 (SE = 0.07) (Figure 5B). At this point in sampling, it was not possible to conclude with statistical confidence that the OTU_0.20_ memberships were significantly different, since both CIs included 1.00; however, we expect further sampling to make the estimates more precise.

## Discussion

In the 20th century, the view of soil microbial ecology shifted from being described by Selman Waksman as a “clear picture” [34] to E. O. Wilson's pronouncement that its diversity is “beyond practical calculation” [35]. We have shown that neither view is wholly correct, but that a confident estimate of bacterial richness is attainable using a set of parameters that have a reasonable biological basis. We have shown that it is possible to obtain an OTU_0.03_ richness estimate for soil for considerably less effort than is required to shotgun sequence a bacterial genome (assuming ~100,000 sequence reads per genome and one to five reads for each of 17,000 16S rRNA gene fragments). Determining the richness of specific phylogenetic groups using lineage-specific PCR primers would further reduce the required effort.

Our analysis can also be applied to guide the design of functional and sequence-based metagenomics projects [36]. Tringe et al. [23] estimated that more than 2 × 10^9^ bp of sequence from 3 × 10^6^ sequence reads would be necessary to obtain 8-fold sequence coverage of the most abundant species in their soil sample assuming a genome size of 6 Mbp. To sequence 8-fold coverage of the most abundant OTU_0.03_ from the simulated Alaskan soil community, approximately 450 genome equivalents, or 3 × 10^9^ bp, would need to be sequenced from the Alaskan soil. To sequence 8-fold coverage of the ten most abundant OTUs_0.03_ from the simulated Alaskan soil community, approximately 1,600 genome equivalents, or 10^10^ bp, would need to be sequenced. Although this amount of DNA may be beyond our current sequencing capacity, the 10^10^ bp is approximately the content of a 275,000-clone fosmid library. Such a library could be easily constructed and would be useful for functional metagenomic approaches. Although not currently feasible, sequencing 8-fold coverage of every OTU_0.03_ in the Alaskan soil metagenome would require sequencing 950,000 genome equivalents or 6 × 10^12^ bp of DNA. Although PCR bias may affect the true community distribution, these values are a helpful guide when designing metagenomics-based experiments. For some groups of organisms, the 3% cutoff between 16S rRNA gene sequences has been found to correlate with 70% similarity between genome sequences; therefore, it is unclear how many contigs would assemble for the predicted level of sequencing effort given the substantial intragenomic variation that may exist between members of the same OTU_0.03_.

Estimating richness does not provide the identity of each bacterial type; in the Alaskan soil we studied, identifying every one of the 5,000 different types of bacteria would require sampling more than 480,000 sequences. Furthermore, our analysis assumes an operational species definition of a group of 16S rRNA sequences that are no more than 3% different from one another. Among the members of a single OTU, there is undoubtedly considerable phenotypic and genomic diversity that is not reflected by 16S rRNA sequences [24]. Our attempt to perform a census of the number of bacteria in a gram of soil provides a guidepost from which we can begin to assess the effects of environmental perturbations on community composition, diversity, evenness, and richness. Moreover, an accurate census would quantify the part of the microbial community that is not accounted for in the current models of community structure and function. In the Alaskan sequence collection, two sequences belonging to the sparsely sampled candidate phylum ACE were found only after sampling 832 sequences. We suspect that members of many poorly sampled candidate phyla are rare members in microbial communities [37], but may play significant functional roles in the microbial community. Although a reliable estimate of richness will inform the development of a conceptual framework for describing the functional biology of the soil microbial community, we will not know the texture and composition of that richness until we have exhaustively sampled and identified every member of the community.

## Materials and Methods

### Clone library construction, sequencing, and analysis.

We obtained a soil core from the Bonanza Creek Long-Term Ecological Research site approximately 30 km southwest of Fairbanks, Alaska, United States (64° 48′ N, 147° 52′ W) on the site designated BP-1 on an island in the Tanana River [38]. The L1A 16S rRNA gene library was constructed using a single 0.5-g sample of soil. The Bio101 soil DNA kit (Bio101, Irvine, California, United States) was used to extract and partially purify genomic DNA and the sample was further purified using a silica matrix (ExpressMatrix; Bio101) until it was suitable for PCR amplification.

16S rRNA genes were amplified in a single reaction by PCR using primers 27f (AGRGTTTGATYMTGGCTCAG) and 1492r (GGYTACCTTGTTACGACTT) and the products were purified by gel extraction (Qiaex II; Qiagen, Valencia, California, United States). Purified PCR products were ligated into the pGEM-T TA cloning vector as described by the manufacturer (Promega, Madison, Wisconsin, United States) and electroporated into E. coli (DH5α). Positive transformants were inoculated overnight into LB with ampicillin (100 μg/ml) and the culture was used as template for PCR using the universal M13f and M13r vector primers. These PCR products were purified using AmpPure (Agencourt Bioscience, Beverly, Massachusetts, United States) and sequenced using the 27f and 787r (CTACCRGGGTATCTAAT) primers. If the 787r primer did not produce quality sequence, we used either the M13f or the M13r primer for sequencing. Sequencing reactions were performed using BigDye version 3.1 (Applied Biosystems, Foster City, California, United States) and were analyzed at the University of Wisconsin-Madison biotechnology center. All clones had 2-fold sequencing coverage for the first approximately 700 bp of the 16S rRNA gene.

Sequence contigs were constructed using STADEN [39] and aligned using ARB [40] with a reference database of more than 16,000 sequences longer than 1 kb. Putative chimeric sequences were identified using Bellerophon [41] and were further screened using CHIMERA_CHECK [42], partial treeing, and comparing the sequence alignment to predicted secondary structure to detect changes in helical base pairing and nucleotide signatures [43]. Phylogenetic placement of the 1,033 sequences was determined by identifying the phylum to which each sequence showed affinity after adding sequences to the database tree using the parsimony algorithm implemented in ARB with a 50% consensus mask.

We also obtained (from Susannah Green Tringe) two 16S rRNA gene sequence collections (*N*_AKYG_ = 875 sequences; *N*_AKYH_ = 758 sequences) constructed using a single 0.5-g sample of Minnesotan (Waseca County, Minnesota, United States [23]) farm soil. The original soil genomic DNA was obtained by cell fractionation followed by enzymatic and chemical extraction of the DNA [23,32]. Since our preliminary analysis using a nonparametric estimator of the fraction of shared OTUs [44] showed that the two Minnesota soil libraries harbored more than 68% of each others' OTU_0.03_ membership, and they were made from the same soil sample but different PCR reactions, we pooled the 1,633 sequences into a single dataset. For direct comparison, the Minnesotan and Alaskan sequence collections were realigned using the NAST aligner [45] at the greengenes Web site (http://greengenes.lbl.gov), and the nucleotide sites between positions 150 and 700 (E. coli numbering) were used in subsequent analyses.

### Community analyses.

To describe the community structure of each soil we used DOTUR's implementation of the furthest neighbor algorithm [18] to assign sequences to OTUs after exporting a Jukes-Cantor corrected distance matrix constructed in ARB using unmasked sequences. Output files from DOTUR were used to calculate collector's curves for the nonparametric estimators of the fraction of sequences from one library that affiliated with the OTUs shared between the libraries [44].

### Model community analysis.

Using a truncated lognormal, Pareto, or uniform distribution, we were able to construct model communities in which the probability of drawing an individual species followed a defined distribution. To identify the most appropriate truncated lognormal distribution that described the observed data, we first selected reasonable values for *μ* between 6.000 and 12.00 and *S*_T_ between 1,000 and 10,000. Next, using Equation 1, we identified values of *σ* that would yield *N*_T_/*N*_max_ values of 10, 35, 40, 45, 50, 100, and 1,000. *N*_T_/*N*_max_ is the reciprocal of the probability observed at the distribution's mode, where *N*_T_ represents the total number of individuals in a community and *N*_max_ represents the abundance of the most abundant member in the community. The values of *N*_T_/*N*_max_ shown in Table 1 were selected because they fell within the range suggested by Curtis et al. [13] for microbial communities and because they resembled the frequency data observed from the Alaskan soil collection. For a given value of *N*_T_/*N*_max_, increasing *μ* increased the value of the reciprocal of the Simpson's index (1/*D*). 1/*D* represents the number of uniformly abundant OTUs needed to observe the same level of diversity found in the community. Using these parameters, we drew random values from the desired lognormal distribution by first drawing a random normal variable with mean *μ* and standard deviation *σ*. Random lognormal variables were then obtained by determining the integer value of *e* ^X^, where X is the value of the random normal variable. Values larger than *S*_T_ were discarded, resulting in random variables drawn from a truncated lognormal distribution. Random variables drawn from either Pareto or uniformly distributed communities were done in an analogous manner.

As random values were generated, we constructed collector's curves for the observed richness and the full bias-corrected Chao1 [19], ACE [20], and interpolated Jackknife [21] nonparametric estimators. The heuristic search did not include the Jackknife estimator because the estimates were highly variable and uninformative. As measures of diversity, we determined 1/*D* and the longest string where duplicate members of the same taxa were not observed. We determined the CI for each metric as a function of sampling effort by constructing 1,000 model communities for each set of model parameters. The sampling of the truncated lognormal distributions and parameter calculation was performed using a C++ computer program that we wrote. If the collector's curve for the sampling of the Alaskan or Minnesotan 16S rRNA sequence collections crossed the CI for any parameter, the simulated community was rejected.

## Supporting Information

### Accession Numbers

The GenBank (http://www.ncbi.nlm.nih.gov) accession numbers of the Alaskan 16S rRNA gene sequences are AY988608 through AY989640. All sequence alignments are available from the authors' Web site (http://www.plantpath.wisc.edu/fac/joh/soil_census_data.html).

## Abbreviations

CI: 95% confidence interval
OTU: operational taxonomic unit
RFLP: restriction fragment length polymorphism
SE: standard error
